# Healthcare professionals’ perspectives on mental health service provision: a pilot focus group study in six European countries

**DOI:** 10.1186/s13033-020-00350-1

**Published:** 2020-03-06

**Authors:** Sofia Triliva, Spyridoula Ntani, Theodoros Giovazolias, Konstantinos Kafetsios, Malin Axelsson, Claudi Bockting, Ann Buysse, Mattias Desmet, Alexis Dewaele, Dewi Hannon, Inger Haukenes, Gunnel Hensing, Reitske Meganck, Kris Rutten, Viktor Schønning, Laura Van Beveren, Joke Vandamme, Simon Øverland

**Affiliations:** 1 Department of Psychology, University of Crete, 74100 Rethymno, Crete, Greece; 2 Department of Care Science, Faculty of Health and Society, Malmö University, Malmö, Sweden; 3 Department of Psychiatry, Amsterdam UMC, Location AMC, University of Amsterdam, Amsterdam, The Netherlands; 4 Institute for Advanced Studies, University of Amsterdam, Amsterdam, The Netherlands; 5 Department of Experimental Clinical and Health Psychology, Faculty of Psychology and Educational Sciences, Ghent University, Ghent, Belgium; 6 Department of Psychoanalysis and Clinical Consulting, Faculty of Psychology and Educational Sciences, Ghent University, Ghent, Belgium; 7 Research Unit for General Practice, NORCE-Norwegian Research Centre, Bergen, Norway; 8 Department of Global Public Health and Primary Care, University of Bergen, Bergen, Norway; 9 Department of Public Health and Community Medicine, Institute of Medicine, The Sahlgrenska Academy at the University of Gothenburg, Gothenburg, Sweden; 10 Department of Educational Studies, Faculty of Psychology and Educational Sciences, Ghent University, Ghent, Belgium; 11 Division of Mental and Physical Health, Norwegian Institute of Public Health & Department of Psychosocial Science, University of Bergen, Bergen, Norway

**Keywords:** Health professionals, Mental healthcare gap, Europe, Optimal mental healthcare

## Abstract

**Background:**

The mental healthcare treatment gap (mhcGAP) in adult populations has been substantiated across Europe. This study formed part of MentALLY, a research project funded by the European Commission, which aimed to gather qualitative empirical evidence to support the provision of European mental healthcare that provides effective treatment to all adults who need it.

**Methods:**

Seven focus groups were conducted with 49 health professionals (HPs), including psychologists, psychiatrists, social workers, general practitioners, and psychiatric nurses who worked in health services in Belgium, Cyprus, Greece, the Netherlands, Norway and Sweden. The focus group discussions centered on the barriers and facilitators to providing quality care to people with mild, medium, and severe mental health problems. Analyses included deductively and inductively driven coding procedures. Cross-country consensus was obtained by summarizing findings in the form of a fact sheet which was shared for triangulation by all the MentALLY partners.

**Results:**

The results converged into two overarching themes: (1) Minding the treatment gap: the availability and accessibility of Mental Health Services (MHS). The mhcGAP gap identified is composed of different elements that constitute the barriers to care, including bridging divides in care provision, obstacles in facilitating access via referrals and creating a collaborative ‘chain of care’. (2) Making therapeutic practice relevant by providing a broad-spectrum of integrated and comprehensive services that value person-centered care comprised of authenticity, flexibility and congruence.

**Conclusions:**

The mhcGAP is comprised of the following barriers: a lack of funding, insufficient capacity of human resources, inaccessibility to comprehensive services and a lack of availability of relevant treatments. The facilitators to the provision of MHC include using collaborative models of primary, secondary and prevention-oriented mental healthcare. Teamwork in providing care was considered to be a more effective and efficient use of resources. HPs believe that the use of e-mental health and emerging digital technologies can enhance care provision. Facilitating access to a relevant continuum of community-based care that is responsive coordinated and in line with people’s needs throughout their lives is an essential aspect of optimal care provision.

## Background

Mental health challenges contribute significantly to psychological, occupational and social burdens. They contribute to morbidity across Europe and are an urgent health priority in countries around the world [1]. Improving access to mental healthcare and mental health literacy is the mission and fundamental goal of both the World Health Organization [2] and the common European Union Health program. This is articulated in the Mental Health Action Plan for Europe (MHAPE) [3]. These directives and goals are based on the principle of equity in terms of access to and availability of healthcare services [2]. The strategic goals outlined in these documents include community-based care that is provided by multidisciplinary teams in a timely, effective, and respectful manner.

The World Health Organization’s (WHO) and the European Commissions’ directives disseminate evidence-based health information that is of importance for stakeholders in the healthcare field. For HPs, guidelines include working in community service settings in partnership with the different sectors of health provision to improve service-users’ access to prevention, health promotion, treatment/intervention and rehabilitation/recovery in order to meet people’s mental, physical, and social needs [2]. Indubitably, for the provision of such comprehensive, integrative, evidence-informed, and effective services that address inequalities and discrimination, a sufficient, well-trained, interdisciplinary workforce of HPs is a necessity. Moreover, HPs and Mental Health Services (MHS) require ample support via sustainable funding and system-level collaboration and organization that enables the forging of partnerships with all sectors of health and social care [3]. The availability of the resources (financing, organizational capacities, human capital and capabilities) required to deliver services in light of the population’s ever-growing mental healthcare (MHC) needs and demands have created a mhcGAP [4].

Albeit MHC systems across Europe follow the guidelines and principles of the WHO and the MHAPE, they vary significantly in how they address the mental healthcare gap (mhcGAP). A recent article based on data from the REFINEMENT project (Research on Financing Systems’ Effect on the Quality of Mental Health Care in Europe) compared mental health systems (MHS) with public coverage in specific cities or districts in eight (Austria, England, Finland, France, Italy, Norway, Romania, and Spain) European countries [5]. The researchers found variability in MHC service availability and capacity in the countries studied. The organization of services differed in the eight countries studied, varying from community-based services to psychiatric hospital-based care. Furthermore, dissimilarities in nomenclature create confusion because the terminology used to describe the services provided does not always reflect the real nature and implementation of services. For example, in Finland, where the MHC system is considered to be community-focused, the pattern of care was found to be hospital-based in reality. Furthermore, in countries administering a variety of services, barriers to care exist due to fragmentation and inter-sectoral collaboration difficulties [5].

In another REFINEMENT project study that mapped the MHS delivered in southern and northern Europe by comparing service structure, personnel capacity, and productivity (using the ‘Description and Evaluation of Services and Directories in Europe for long-term care’), the researchers found similarities in the public coverage provided in the southern and northern regions of the continent that had analogous systems of care provision. However, there were differences in emphasis whereby the system in the northern region was hospital-focused and in the southern region community-focused [6]. Additionally, there were considerable differences in personnel capacity between the regions, with physicians, nurses, psychologists and other HPs in the northern regions displaying much greater workforce capacity. Moreover, disparities between the two regions were reported in MHS productivity, with southern European regions serving greater numbers of people in population-adjusted comparisons. According to Sadeniemi et al. [6] these noteworthy differences reflect historical and cultural divergences. For example, strong family networks and ties in southern Europe any regions are associated with a lesser need for and use of hospital-care and residential facilities and to “trans-institutionalization to non-hospital residential services” (p. 10). The REFINEMENT researchers identified the need for further studies that can inform policy and context-specific analysis in order to develop an in-depth understanding of accessibility and effectiveness in MHS provision in different countries across Europe.

The gap between MHC needs and MHS provision has prompted evaluative research in several European countries, including Turkey, England, and the Netherlands [7–9]. The evaluation of care provision from HPs using qualitative methodologies identified positive perspectives regarding newly developed community mental health centers in Turkey. A need for improvement in the following areas was also recommended: dealing with social stigma; greater integration of services; HPs’ training; and the application of holistic person-centered services [7]. Similar recommendations were made by Weinmann and Koesters [10] in their review of the literature on MHC in low and middle-income countries. Their study described the accessibility gap and the shift from hospital-based to community-based MHS. The subsequent recommendations included training HPs working in primary care, community rehabilitation, task-shifting as a means to increase capacity, and the delivery of culturally attuned services. In order to close the availability gap and to reduce social stigma, people with severe mental health needs are often served in primary healthcare. In a study that focused on such MHC arrangements, Beckers et al. [8] report that such needs can be effectively served in primary care when professionals are mindful of personal recovery goals, use social supports and have adequate training. Patel and Chatterji [11] and Javadi et al. [12] recommended comprehensive training, teamwork, and task shifting to increase the capacities of HPs working in primary care as ways to enhance and augment the effectiveness of person-centered care.

In a similar vein, Montgomery et al. [9] evaluated MHS provision in Northern Ireland using qualitative methods, namely, in-depth interviews with HPs and service-users. Their analyses revealed improvements in that there has been a shift from long-term hospital care to community-based care and person-centered approaches. The need for more integrated and better coordinated services, as opposed to fragmented services with poor inter-sectoral collaboration, was underscored. There was an emphasis on shifting from the medical to the recovery model of care provision. Funding problems due to budgetary constraints and the prioritizing of physical health as well as improving service-user involvement in decision-making were also highlighted. The authors made recommendations to carry out further research focusing on how to implement more effective services that are person-centered and based on “recovery ethos” (p. 105).

In chime with the above, Barbato et al. [13] report that access to MHC in Europe differs among countries and that overall there is a treatment gap for all mental health problems, especially for more common mental health needs such as anxiety and depression. Moreover, according to the same authors [13] people more usually receive ineffective treatment. In addition, care does not guarantee treatment adequacy and the “minimally adequate treatment” is essentially below the threshold of sufficient care (p. 15).

Given the crucial role of the social determinants of health play, as well as the significance of holistic care that includes biological, psychological, and social supports and services that are readily accessible for people who need them, the WHO includes the following in their definition of optimal MHs: Optimal MHS here refers to the provision of comprehensive supports and services that are easily accessible within the community. Such services include self-care and informal community care, primary care, community mental health services, psychiatric services in general hospitals, and long-stay facilities and specialist services. The WHO organization underscores that public health systems should use evidence-based and emerging practices that are community-based, driven by service users and their families or networks and that include self-determination and informed decision-making by service users [3].

De Silva et al. [14] carried out a literature review of the different methods of evaluating the coverage of psychological treatment, concluding that there are very few studies focusing on evaluating effective and optimal coverage. These researchers stressed the need to develop a comprehensive understanding of how MHC is administered across Europe. The difficulties involved in evaluating and unpacking the barriers and facilitators when it comes to implementing the provision of complex health services require multidisciplinary approaches that include communication with multiple stakeholders in order to gain broader and more detailed knowledge of the processes, means, and impact of services on people’s lives and concomitantly, the work lives of both professionals and service-users [15]. Heeding attention to these complexities, the present study aims to contribute to the literature by developing a more in-depth understanding of the advantageous and disadvantageous factors in MHC service provision in six countries: Belgium, Cyprus, Greece, the Netherlands, Norway and Sweden.

The study was part of MentALLY, a pilot project funded by the European Commission to gather qualitative empirical evidence in order to establish European mental healthcare that provides effective treatments to all adults who need it (http://mentally-project.eu/). This study aimed to answer the following questions:

1. What are health professionals’ perspectives on the barriers and facilitators to implementing accessible and effective MHC?
2. What practices and skills do HPs working in MHS consider important and necessary in facilitating accessibility, referral practices, collaboration and positive treatment outcomes?

## Methods

The goal of this qualitative research was to explore and critically analyze the core positive and negative aspects of mental healthcare provision across the six aforementioned countries.

### Design of the study

The research was overseen by the MentALLY research consortium—a multidisciplinary team of researchers who actively participated in the design, implementation, and analysis of the results of the study. Qualitative research procedures were used in data collection and in the analysis of the findings in an effort to foster understandings about MHC service provision and to ultimately identify ways to support the promotion of optimal MHS delivery across Europe.

Focus group methodology was used in the generation and collection of the data. A qualitative approach was preferred as it is a strategy that engages research participants in dialogue and social interaction, stimulating meaning-making at both the individual and group levels [16]. The intention of the semi-structured focus group interviews was to generate natural discussions amongst the HPs in each of the MentALLY countries. Another purpose of these interviews was to cultivate research circumstances to discuss and understand HPs’ insights and perspectives into the accessibility of MHC and effective or ineffective practices in the MHC settings where they work.

### Ethics and procedures

The research protocol was approved from the institutional review boards of Ghent University and the University of Crete, the Regional Ethical Review Board of Gothenburg, the Norwegian Regional Council of Medical Ethics, the Medical Ethical Committee of Academic Medical Hospital of the University of Amsterdam and the Mental Health Services office of the Ministry of Health of Cyprus. Data collection took place between April and November 2018 in all of the MentALLY countries. Written informed consent to audio-record, anonymously report and publish the research data was provided by all participants who took part in the focus groups. The data was collected from seven focus group discussions with HPs. One focus group was conducted in each of the following countries: Belgium (the Flanders region), Cyprus, Greece, the Netherlands and Norway, and two in Sweden.

### Participants

Each focus group consisted of 3 to 11 participants who were recruited according to the following criteria: professional background (including psychologists, psychiatrists, general practitioners, social workers, nurses and physiotherapists), service sector (i.e. public or private practice, working within the confines of an interdisciplinary team or independently), and gender (an attempt to include an equal number of men and women in each group). Participants were recruited by using snowball sampling through the MentALLY teams’ contacts who specialized in primary and secondary healthcare. In order to increase the likelihood that the subsequent connections in the snowballing process could reach diverse groups of mental health professionals who were willing and eligible to participate, we started with a very diverse network of initial informants. By using this variation in maximum diversity sampling selection bias was minimized.

Participants were informed of the study by phone or e-mail and provided with information about its goals, the procedures that would be followed and the voluntary basis of their participation. Interested healthcare professionals were contacted by one researcher from each team and given detailed information regarding the time and location for the focus group. The participants’ work affiliations and professional backgrounds are presented in Table 1.

**Table 1:** Participants’ professional title, service setting, and country

Country	Psychiatrists		Psychologists		GPs		Psychiatric nurses, social workers and physiotherapists	
	Number	Work setting^a^	Number	Work setting	Number	Work setting	Number	Work setting
Belgium	2	PH and private practice PH	3	General hospital and private practice PH Outpatient MHC	2	National organization for refugees Private practice	2	Center for general well being Community health center
Cyprus	2	MHSs, rehabilitation clinic Private psychiatric clinic	4	MHSs, day center Private psychiatric clinic Community mental health department, outpatient services Private practice	1	General hospital	1	Public PH Community service
Greece	4	Public hospital Psychiatric inpatient and outpatient unit Public hospital, psychiatric department Private practice (2) Collaboration with public MH services	4	Short-term hospice service unit/after hospitalization care Private psychiatric clinic Private practice (2) NGO	2	PHC (2)	1	Public hospital Psychiatric Unit
Norway	–	–	2	PHC Substance abuse treatment/private practice	2	General practitioner Nursing home	1	Outpatient clinic
Sweden	3	Outpatient care (addiction) Specialized Psychiatric care at a hospital (psychosis) PH (affective disorders)	2	Psychiatry, in- and outpatient departments PH (affective disorders)	2	PHC (2)	1	Rehabilitation center
The Netherlands	3	PH (2) Academic hospital, Department of psychiatry	3	MHC (3)	2	University hospital, Department of Geriatric Medicine and General Practitioner Medicine General Practice	–	–
Total participants	14		18		11		6	

^a^Some participants may work at more than one setting

*PH* psychiatric hospital, *MHSs* mental health services, *PHC* Primary Health Center, *MHC* Mental Health Center

As is evident from Table 1, the 49 focus group participants included psychologists, psychiatrists, social workers, general practitioners, psychiatric nurses, and other health professionals. The HPs worked in both public and private psychiatric hospitals, general hospitals, NGOs and private practice. The diversity in the HPs’ specialization along with the number of participants broadened the range of thoughts, opinions, and ideas expressed.

The HPs participated in focus group discussions that were conducted by experienced moderators-researchers of this study and followed the same thematic guide comprised of a set of open-ended questions translated into each country’s spoken language. The focus group discussions evolved around the following themes: (1) Description of mental healthcare provision focusing on positive and negative experiences; (2) Accessibility and availability of adequate and sufficient mental health services; (3) Knowledge required of HPs for assessment and referral; (4) Treatment, in terms of the knowledge and skills HPs need to acquire in order to bring about the optimal treatment outcomes; and (5) Collaboration: how can HPs working in and outside of MHC coordinate to provide optimal care. Interview prompts were based on the themes that emerged in the discussion. The audio recorded focus group discussion times ranged from 90 and 141 min. The discussions were transcribed verbatim by the researchers, anonymized and were either analyzed in the language they were recorded or translated into English (by the researchers who were either native or fluent English speakers) prior to analysis.

### Analyses

Analyses were conducted by applying thematic analysis (TA) procedures [17–19]. TA was applied in order to identify patterns of meaning across the dataset for each country. The analysis followed the step-by-step procedures presented in Brown and Clarke [17, 19] aiming to identify themes which were interesting and important to our research questions. The data were analyzed deductively and inductively by two or three members of the teams from Belgium, Sweden, Norway and Greece. Data from the Netherlands were analyzed by the Belgian team and data from Cyprus by the Greek team. The analysis resulted in a series of main themes and subthemes for each country, which were presented in factsheets that included the resulting themes and the quotes supporting them. In this manner, the factsheets reflected interpretations and sense-making that were more closely aligned with the culture of participants in each country and the reflexivity of the researchers.

The TA process involves familiarization with the data, data reduction through initial coding (both theoretically and inductively driven), search for themes (i.e., meaningful patterns related to the research questions), review of themes (search for overlapping or relevant themes, support of themes by the data, repetition of themes, search for new themes, and so forth), definition of themes (refinement of the themes, description, identification of subthemes, relevant quotes), obtaining consensus and writing up fact sheets to be shared by all MentALLY partners. The MentALLY team discussed and deliberated regarding the themes via presentations in face-to-face and online meetings and by collaborating on compiling and triangulating the analyses. They were weaved together to summarize and make sense of the data into a narrative representing the six MentALLY partners’ sharing and recounting the delivery of MHS in their respective countries.

## Results

**Fig. 1:**
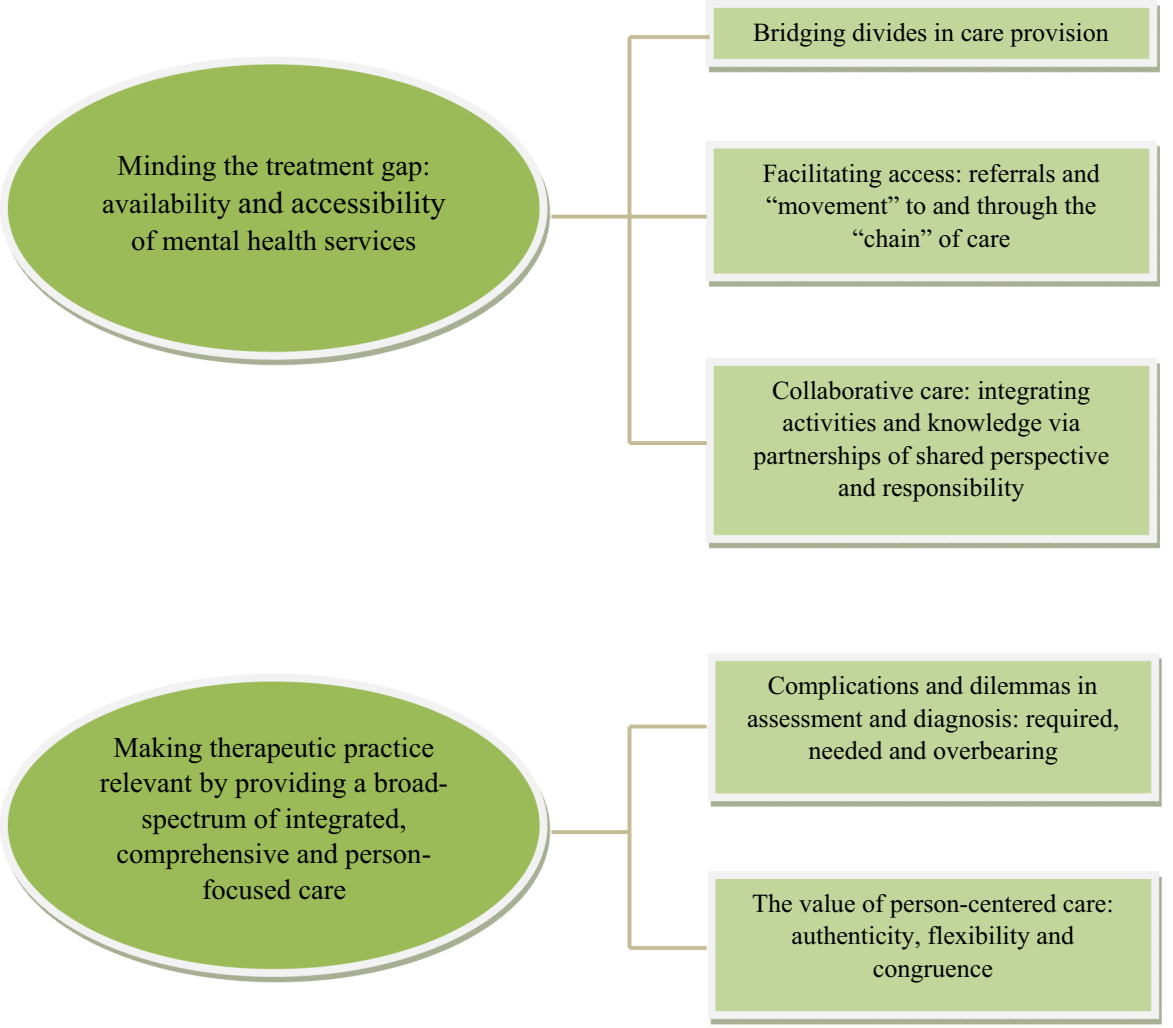
Themes and subthemes emerging from TA analysis

The results converged under two overarching themes: (1) Minding the treatment gap: the availability and accessibility of MHS. The gap is composed of different elements that constitute barriers to care, including bridging divides in care provision, obstacles in facilitating access via referrals and creating a collaborative ‘chain of care’, (2) Making therapeutic practice relevant by providing a broad-spectrum of integrated and comprehensive services that value person-centered care comprised of authenticity, flexibility and congruence. Figure 1 summarizes the overarching themes and the subthemes resulting from the data analysis. The discussion below focuses on these thematic clusters.

### Minding the treatment gap: availability and accessibility of mental health services

#### Bridging divides in care provision

The HPs in the six countries highlighted the discrepancy that exists between the ever-growing numbers of people needing MHC and those obtaining access and receiving care, as well as the importance of minding this gap. According to the HPs, there are several barriers and obstacles to the availability and accessibility of MHC. In Norway, access to MHC is described as a process of accessing a *“castle surrounded by a moat”.* This metaphor is fitting for all of the MentALLY countries with variations as to the determinants of this gap. In Cyprus, Health Services (HS) have undergone a severe economic crisis to the detriment of quality and equitable accessibility to MHS. In Greece, a long process of de-institutionalization and expansion of community services is still in progress. Hence, publicly funded services are available (mostly for severe psychiatric problems) but not always accessible due to understaffing (especially psychologists) and funding problems. In the Netherlands, there are capacity problems for those with chronic conditions as well as crisis care. The HPs in the Netherlands attribute these barriers to a focus on primary care service provision alongside a shrinking secondary care sector. Both in Belgium and Sweden, HPs stress that people seeking MHS do not get the necessary help when they need it due to waiting lists. Inadequate funding and staffing shortages are considered common barriers to providing quality services in all of the countries. In addition, social stigma continues to be a barrier. The following excerpts are indicative of the perspectives expressed in the focus groups regarding the provision of readily accessible MHC.

*“I think the specialist health service has grown to be something like a large fortress with a big moat around it, and you need to work pretty hard to gain access, but as soon as you are in there, a lot is happening and then you are back out again. There are some artificial distinctions in this, and I have to admit, that as a psychologist I think that all outpatient clinic services should take place in primary healthcare and that people could just show up there.”* (HP, Norway)

The mhcGAP is also comprised of barriers in how services are organized. The HPs recommended integrating primary care and specialized outpatient services in order to optimize care provision and to fortify collaborative practices. According to the HPs, fragmented services and the internal organization of HS lead to difficulties in care provision and in service-users’ and HPs’ satisfaction.

*“The different (MHC) institutions are too isolated and ask a bit too much either of this or that. Then there is the problem with the organization of the health services. The structuring, coordination and patient flow…There are too many areas where we fall short.”* (HP, Norway)

In Greece, accessibility is also hampered by geographical barriers, impediments in developing comprehensive services, and the reimbursement of psychotherapeutic treatment. The excerpt below details ways to span such gaps.

“*Accessibility is vital. Also, when we say accessibility, we do not mean just how easy it is for someone to come to us, but also for us to go and find them where they are…to follow*-*up. There are a variety of services that should be available in ‘good mental health services.’ Accessibility also means…to have continuity in therapy, people who act as a point of reference need to be consistently available and service*-*users should not change providers all the time.”* (Psychiatrist, Greece)

In all six MentALLY countries, there was an emphasis on the growing inequality fueled by accessibility barriers, long waiting lists and a lack of readily accessible specialized care. These impediments were enumerated and analyzed in all of the focus group discussions and are in line with a lack in availability of comprehensive treatment, especially in rural areas of Greece, Norway and Cyprus. In Sweden, Belgium, Norway and the Netherlands, MHS are partially integrated into primary care as a means of narrowing the treatment gap. Nevertheless, barriers with referrals and follow-up services persist as elements that present difficulties in providing ‘optimal’ or even ‘sufficient’ care.

HPs in Belgium described the ‘*unfairness’ and ‘inequity’* of the mental healthcare systems they serve. The systems are ‘*unjust*’ because some people do not receive care at all (e.g., due to a lack of affordable or publicly funded services), others do not receive the right kind of care (e.g., medication instead of psychotherapy or primary care as opposed to specialized care) and others receive care but have to endure long waiting times. The excerpt below is a vivid description of the difficulties in accessibility for service-users who seek and need immediate help.

*“I have never heard a surgeon say ‘oh they have appendicitis. Well, I don’t have time for that so, they can wait’. With crisis care, I kind of get that sense. You have to visit them and give several arguments for the patient’s case… These patients need help, quick. There shouldn’t be any waiting lists for these kinds of patients…the severely suicidal patients that need immediate help but can’t find the help.”* (General Practitioner, the Netherlands)

The participants emphasized the divide between physical and mental healthcare and the wider gap in availability and accessibility for mental healthcare in different ways. They described how social stigma and self-stigma contribute to the gap. For example, in Cyprus, participants discussed at length how the prevalence of mental health stigma in the Cypriot society impacts peoples’ decision-making regarding seeking MHC. As the HPs from Cyprus stated people seeking MHC prefer to travel long distances to visit a facility where they do not want the risk of being seen by people they know and this is not the same for physical healthcare. HPs expressed apprehension regarding stigmatization and discussed ways to confront it, as described in the excerpt below.

“*In the hospitals, there should be many psychiatric beds which should not be isolated from the rest of the hospital as this leads to the stigmatization of the mental health patients*…*What brings us better accessibility is de*-*stigmatization, and I believe that this is an area where the state should fund programs for the de*-*stigmatization of mental disorders*.” (Psychiatrist, Cyprus)

HPs from Sweden explained that the system of care in their country is based on a scoring system that takes into account the number of service-users and diagnoses, with less compensation allotted to those confronting mental health problems.

*“You can’t run healthcare like you can run a business. Because business is completely different, and care has to cost money…The patients whom you get paid least for are those with mental illnesses, so they are hit the hardest because you can’t spend the need with them*” (HP, Sweden).

The HPs in all the focus groups were mindful and concerned about these obstacles in accessing care. The idea of an “*easily accessible first line psychologist*” (Belgium) who can immediately assess needs and who can refer patients to appropriate and immediate help was a focus of the discussion in all the MentALLY countries.

#### Facilitating access: referrals and ‘movement’ to and through the ‘chain’ of care

The HPs that took part in the study accentuated the need to establish and reinforce primary care (PC) with services that are organized to facilitate access. They underlined the importance of PC in referral processes and the need for well-outlined guidelines for referral practices in order to foster timely and high-quality mental healthcare. There appear to be gray areas in how referrals can best be handled, and HPs are often hard-pressed to draw the line regarding the scope of their practice in managing service-users’ concerns versus making a referral. Moreover, “*referrals are often lost between different services”* (e.g., primary care and specialized care in Sweden). HPs have difficulty “*in identifying mental health providers and the available services”* in some of the MentALLY countries (HP, Belgium).

In Sweden, referral processes were dubbed ‘*risky for patients*’ and referring patients to psychiatric care was described as a complicated and time-consuming challenge. They described that patients waiting to be referred to psychiatric care suffered greatly. The participants in the focus group shared their experiences:

“*The referrals to MHC are often returned. And then I write a new referral, and another referral and they are seriously ill. Referrals become a game between primary and psychiatric care to the detriment of the patients.”* (HP, Sweden)

The HPs noted that facilitators in MHS provision include having a broad spectrum of services that operate as *“a chain of care”* on the basis of *“blind trust”* (Belgium) between providers, service-users and the wider service system. Yet, establishing such a chain is not always possible, especially where primary care is in a neophyte stage of development (Greece, Cyprus). In Norway, on the other hand, referrals and “*the movement of patients”* between different branches of mental healthcare were described as “*halted by heavy bureaucratic processes*”. Difficulties in referral between the diverse forms of care were also mentioned (Sweden and the Netherlands).

*“I do not always get a response on the referrals I send, so I do not know whether these people receive an offer or whether they are now in nowhere land. This makes me a bit uncertain about referrals, and it might raise the threshold for referring. But I always try to refer of course, as I do not want to rob them of any rights, but it is challenging.”*(HP, Norway)

The HPs in this study agreed that referrals are a core component in the delivery of mental healthcare. Yet, missing replies on referrals, unanswered e-mails, or difficulties in reaching colleagues providing specialized services were frustrations mentioned by HPs in Sweden, the Netherlands, Belgium and Norway. In Greece and Cyprus, the “*absence of a referral culture*” was discussed at length. Cognizant of the mhcGAP the participants, noted that appropriate referrals require the establishment of open communication channels, partnerships in care provision, matching service-users to appropriate services, and helping service-users to accept, engage, and benefit from services.

The HPs pointed out that the health systems they serve are under a great deal of strain due to a confluence of factors including the high demand for services, staff shortages, and communication difficulties between HPs as well as with service-users. These factors vary in the different six countries, still, they hinder MHS provision because referral, communication and collaboration processes falter between the different sections of the pyramid of care, impacting services users’ “movement” in the systems of care.

#### Collaborative care: integrating activities and knowledge via partnerships of shared perspective and responsibility

The countries involved in the MentALLY project provide a broad spectrum of MH services, including MH promotion, prevention, early identification of MH concerns, referrals for specialized treatment and different forms of therapy in public and private settings. Nevertheless, HPs emphasized the “*fragmentation of existing forms of care”* (Belgium, Norway) and the “*parallel and conflicting operation of different forms of care”* (i.e., between primary care and specialized care in Sweden).

In the Netherlands, participants stated that “*MHC is influenced by private market*-*mechanisms”* (insurance companies who only reimburse specific forms of treatment). Due to this, healthcare providers “*pick the ‘easy’ people to treat”*, and as a consequence, people with more complex problems needing specialized and long-term care are oftentimes left untreated.

*“The private institutes cherry*-*pick their patients. Like, oh that patient is good, because I can do short DBCs (diagnosis*-*treatment*-*circuits) and have good results. I’ll take those. I hear from several general practitioners that certain patients are denied care by certain larger institutes. The caregivers refer and the institutes say, ‘No, we won’t take this patient’.”* (Psychiatrist, the Netherlands)

Collaborative relationships between primary care physicians and MHC providers require extensive effort and work. In Greece, Cyprus, Belgium and the Netherlands, HPs proposed linking prevention and treatment within an integrated framework in order to provide adequate and comprehensive care. Diverse approaches to the *“prevention and the promotion of mental health”*, including the *“psychoeducation of the general public”* on mental health problems at the primary level were discussed. There is often a lack of availability for secondary prevention, additional treatment or wrap-around services designed to strengthen the therapeutic gains for individuals who did not fully benefit from the standard program or whose recovery seems fragile. *Continuity in care*, which encompasses an array of strategies used in an ongoing way over extended periods to support those individuals diagnosed with persistent, long-term conditions, is considered essential and a means of optimizing MHS. The different levels of service provision and the integration of services in a pyramid-like structure are described in the excerpt below.

“*This is what I am thinking; prevention, treatment, stabilization, psycho*-*education, de*-*stigmatization, and in general, the improvement of the quality of life of the recipients of mental health services.*” (Psychologist, discussing ideal MHC, Greece)

There was widespread agreement among the HPs in the six countries that integrating services in order to deliver better outcomes for beneficiaries entails different levels and types of collaboration. Collaboration between primary and specialized care, inter-sectoral collaboration with other organizations or institutions in order to work on MH related policies, and collaboration with service-users were discussed in all the focus groups. Nevertheless, the collaboration between different services such as public and private services is difficult in some countries (Cyprus and Greece) due to legislative obstacles. HPs stated that collaboration between primary healthcare and specialized healthcare enhances treatment outcomes but is not always feasible (Sweden, Norway). Participants, therefore, made compelling arguments for effective teamwork fostered through collaboration and networking processes aimed at achieving a service user-centered based approach to service provision. Participants in Belgium described obstacles to collaboration as follows:

*“You know that some doctors you can call and that you are always welcome, other doctors find it disturbing… I’m talking about doctors now…yes, with other people you have to e*-*mail or some prefer a letter or… that alone, the communication has struggles, and I experience it as a real problem.”* (Psychologist, Belgium)

HPs talked about collaboration with service-users and applying user-generated knowledge in service delivery (co-production) as something that is important but not always feasible. Co-production implies the forging of positive relationships between service-users and HPs and partnerships in care provision, including carers and family members, by giving people who seek services a role and a say in their care. HPs from Sweden emphasized collaboration and shared decision-making with people in treatment as essential components of their work and described how inter-professional care requires teamwork.

*It is extremely important to plan treatment primarily in consultation with the patient. Teamwork is critical for this.”* (HP, Sweden)

According to the HPs, co-production enforces people’s capabilities by offering people a range of incentives to work in reciprocal relationships with professionals and with each other. If service-users engage with peers, there is a blurring of distinctions, allowing for the reduction of stigma. Service-users’ agency and connection to the community are also facilitated.

Participants in Belgium discussed the ideal society where people cooperate with each other and try to understand each other, stating that MHC should be moving, “*towards a community of caring for each other.”* (HP, Belgium)

Participants also discussed practices that create barriers with service-users. HPs in Greece, Cyprus, and Sweden talked about involuntary commitment and its detrimental impact on service-users and HPs’ relationships.

*“People may leave the hospital thinking: ‘I have received compulsory therapy, I am ok now, and I am leaving, and I do not want to see any MH professional in my life!’ Thus, the patient is lost to the system.”* (Psychiatric Nurse, Cyprus)

Relationships between HPs and teamwork were also discussed. Difficulties in collaboration included “*conflicts”***(**Cyprus) and “*mistrust”* (Belgium and Greece) between HPs. Participants suggested that adequate coordination and meaningful communication between HPs, service-users and consultants in a referral system maximizes MHC’s efficiency and effectiveness.

In addition, HPs explained that collaboration entails practitioners’ *“knowing their limitations and the boundaries of their competencies”* and holding an ethical stance by not engaging in practice beyond these limitations and boundaries. This was mentioned in the focus groups in the Netherlands, Cyprus, and Greece.

*“As a psychologist/psychiatrist, as every caregiver, you should be able to say, I can or cannot do this. And say to the patient, I am going to send you to a specialist and you are going to be treated by them. The first step to do this is being able to say; I do not treat this, it isn’t my expertise. This needs a different mindset.”* (Psychiatrist, the Netherlands)

According to the discussions in all the focus groups, service-users benefit from respectful listening, responsiveness to their expressed needs, and in taking part in decision-making regarding their lives. Overall, HPs recognized that collaborative practices address the problems relating to gaps in services, and can lead to a streamlining of services as well as to improving service-users’ choices and making their voices heard. Likewise, they are hopeful that the systemic incorporation of new information technology in healthcare systems will further improve the communication network among HPs towards achieving the best quality of care.

### Making therapeutic practice relevant by providing a broad-spectrum of integrated, comprehensive, and person-focused care

HPs in all countries involved in the MentALLY project proposed comprehensive models of care where service-users have a say in their treatment, receive the requisite support and can access services that are organized, equitable, and tailored to users’ needs.

According to a recent joint OECD/European Commission report, substantial efforts are required to prevent mental health problems and to establish an effective and efficient early diagnosis and intervention paradigms [20]. Despite the differences that exist in health and mental health service delivery models across the MentALLY countries, such efforts are required in all of the countries. Assessment, early identification and diagnosis issues figured prominently as barriers to ‘appropriate’ service delivery in the focus group discussions.

#### Complications and dilemmas in assessment and diagnosis: required, needed and overbearing

The MH professionals who participated in the focus groups expressed concern that they “*often have to work beyond their professional competencies in diagnostic procedures*” (Belgium, Greece, Cyprus) or they ***“****lack the tools required to provide the best care”* (lack of standardized assessment tools in Cyprus or evidence-based protocols for diagnosis in Greece). HPs in Greece stated that a lack of “*state support in continuing training and education*” (Cyprus), of European-wide licensing, credentialing, privileging and accreditation procedures create diagnostic issues, treatment problems and ethical dilemmas. In Sweden and Norway on the other hand, professionals presented different reasons to the complications in the early identification and diagnosis of people’s mental health needs, namely, the *“chase for a diagnosis”* and “*working in predetermined and inflexible frameworks”* where only one specific model of care can be provided.

In Sweden, where service delivery is based on a system of integrated care, that is, services are provided on a team-based approach, diagnostic and coordination complications and obstacles were discussed.

“*And then I think…while we are talking about primary care, there is a huge fear…in primary care…general practitioners…of psychiatry. There is an awful lot of misdiagnosis there…you could say that there is a real state of chaos that needs to be cleared up. There is a need for training, so that there is accuracy in diagnosis and that diagnosis leads to suitable referrals*.” (HP, Sweden)

Diagnosis obstacles were discussed in all the focus groups with differing foci. In Cyprus, assessment and diagnosis dilemmas are encountered in secondary care.

*“It is usually based on our subjective evaluation as to whether the outcomes we get are good or bad.”* (Psychologist, Cyprus)

Co-occurring acute and chronic health problems and the complex nature of mental health problems, where several life domains are implicated, create further trepidation regarding diagnosis. In Belgium, this complexity was discussed as a barrier to efficient diagnosis, referral and treatment-procedures because services can be over-specialized to the detriment of implementing more holistic and person-centered approaches.

*“Specialized care is where my concern is especially. I work in that setting that has become too specialized, by which you create exclusions because they focus too much on ‘no, we only work on those problems.’ Yes, people are complex creatures, mostly it is not an isolated problem.”* (HP, Belgium)

Participants working in primary care in Sweden described that they felt “*trapped*” in the financial compensation system they have to implement, which leads to “*a chase of diagnoses*”. They dubbed this required labeling as “devastating” because it can be overbearing for people and their lives.

“*There is a huge concern that I am experiencing … in our obsession with diagnosis codes…so I eschew using unspecified mental disorder. Because I think that there are people in a crisis in their lives. For some people, becoming an adult involves a lot of anxiety, but if I were to categorize it and, even worse, send them to mental health care, they could get a personality disorder diagnosis, and perhaps they fulfill the criteria, but is it any use to them…And so I don’t see the benefit in getting a diagnosis. Meanwhile, I must bear in mind that in order to be reimbursed, a diagnosis is necessary. Which I think is totally absurd. It is devastating… so you are forced to write a diagnosis.”* (HP, Sweden)

Thus, the widely held, and often enshrined in numerous policy and practice documents, belief that diagnosis is an indispensable first step to care provision is being questioned with regard to its benefits for service-users.

#### The value of person-centered care: authenticity, flexibility and congruence

According to all of the focus group discussions with the HPs who took part in this study, person-centered care is the optimal and most beneficial approach to delivering MHS. In Norway, participants described the “*flexibility of choosing an appropriate treatment for each patient”* as an essential aspect of their job which, in turn, facilitates their ability to deliver quality services. In Sweden, participants explained that continuity in care is of utmost importance for service-users diagnosed with psychosis. Therefore, a model for continuity in care was incorporated into the treatment plan for such cases.

*“For example, our clinic… psychosis psychiatry…there are people who are responsible for each patient …there are people who organize the care and collaboration around the patients. And that works well, and in some clinics, it works… very well.”* (HP, Sweden)

The participants agreed on the importance of being able to adapt services to the needs of the service-users as opposed to having to deliver a standardized package to every person seeking help.They described the flexibility of choosing a suitable treatment which is of appropriate length for each service user as a fundamental aspect in the provision of optimal care and also their sense of efficacy.

*“I am very happy that I have a framework that allows me to be more flexible for those patients, it is not very fixed with strict instructions on what to deliver and when to finish treatment, and this makes it work well……I often think that what helps me in meeting people in the best possible way is that I have a certain space for autonomy within the framework.…I can allow myself to do those things without someone looking over my shoulder’. I also believe it makes you feel more connected to the work you are doing, that again you make sure you do not go out of your mandate too often either.”* (HP, Norway)

In Sweden, on the other hand, there was talk of ‘reclaiming professional competence’ in that the participants felt that despite their competence and license to practice, the ‘management of primary care’ did not allow them to make clinical decisions based on evidence or best practice guidelines. They described how the management of primary healthcare was organized according to “New Public Management, based on performance and financial values” and noted that these arrangements were not compatible with the provision of optimal MHC.

*“We can sit and talk about a lot of things, but we can’t do anything about this control, we are not allowed to decide ourselves…”* (HP, Sweden)

In Sweden and the Netherlands, the HPs described that due to the management and compensation systems that are in place, they could not offer patients individualized treatment which they considered optimal. The focus group participants championed comprehensive services that include the holistic understanding of peoples’ lives as well as continuity in care.

Another aspect of optimal care was described by HPs in all the focus groups as *‘being authentically there’* and developing a positive and unique long-term relationship or ‘bond’ with the service-users. HPs also emphasized that person-centered care should not be hindered by administrative constraints.MHC can only be adequate when care providers treat people not as objects (diagnoses or parts of their problem) but as people with dignity.

*“I think it is an added value, that you can leave all that labelling behind and just go into a conversation with that person and move on with his question…”* (Social Worker, Belgium)

According to the participants, tailoring psychological treatments to the service-users’ specific needs compliments authenticity and leads to congruence. Relationship-building is considered crucial in care provision. Nonetheless, it takes time and is difficult to accomplish in the high ‘patient flow’ rates in MHC provision, especially primary care.

*“‘Quality of contact’, in the first place, I think that is the best way to gain trust because you cannot enforce trust. You can’t say ‘trust me, I will find the way for you and I will treat you or refer you’. You have to earn it gradually with little continuous steps.”* (General practitioner, Belgium).

According to the HPs, therapy and care practices have to be relevant to people’s everyday lives and collaboration at different levels is crucial in achieving this. Participants stressed how positive collaborative relationships are indispensable when HPs want to fit care practices to the uniqueness of each person’s circumstances, to bolster relationship building and to enhance therapeutic outcomes. Nonetheless, this type of care involves HPs from different disciplines and service units working together to offer integrated services, ensuring that service-users receive appropriate and timely help. The participants maintained that holding all involved parties accountable is challenging. Barriers in collaboration ensue because of the lack of supportive organizational structures, guidelines, and communication that focuses on people’s distress and not on diagnostic nomenclature.

## Discussion

The purpose of the present study was to explore health professionals’ perspectives on the barriers and facilitators to providing optimal MHC and to investigate their views regarding the practices and skills they consider important and necessary in facilitating accessibility, referral practices, collaboration and positive treatment outcomes.

Participants described how optimal MHC provision continues to be an economic and social challenge across the six European countries due to the high prevalence of mental health needs and the apparent gaps in the availability of comprehensive and affordable services, funding and human resources shortages and treatment barriers. The mhcGAP in Europe is substantiated in the research literature as encompassing funding, differential accessibility, capacity and treatment delivery barriers [21–23].

Funding problems include budgetary and policy constraints in providing sufficient and readily available services [13]. Focus groups participants noted such difficulties mostly in Greece and in Cyprus where severe austerity measures in recent years have resulted in healthcare budget cuts [24]. Moreover, increases in MHC needs [25] have rendered access to affordable MHC difficult [26–28]. Socioeconomic disparities in the accessibility and availability of MHC were noted in all of the MentALLY countries, and participants explained how they are attributed to an equity gap [29]. The funding barriers discussed in the focus groups coincide with those discussed in the literature and included the costs and reimbursement of mental health services, policy and legal constraints in providing sufficient and readily accessible services and too few providers [30].

Disparities arise and equity in care is compromised when access to care is not ensured regardless of people’s socioeconomic status, place of residence, gender, race or specific mental health challenges [31]. The participants discussed how to bridge the differential availability gap in services available for people in rural areas, those confronting socioeconomic difficulties, complex health, mental health and social problems, and issues such as cultural/ethnic/language differences and refugee status. These impasses have also emerged in the literature on service delivery for diverse populations [32]. Disparities in MHC provision in rural vs urban areas are attributed to unavailable services, capacity problems in care provision, and social stigma [33]. Another determinant of the mhcGAP is the social stigma which was discussed by the participants in connection to the differences in funding for MH as opposed to other health services [34, 35]. These inequities in accessibility are discussed in the literature and HPs are mindful of them [36]. The HPs concurred with the literature that increases in funding and human resources, along with the application of mobile mental health services, electronic health records, screening tools, and the application of electronic mental healthcare (e-MHC) are seen as positive developments [12] that can diminish the financial problems and inequity in accessibility.

The discrepancies in the availability and accessibility of MHS recounted by the participants in this study are described in the literature as being inherently associated with the prevailing modes of providing psychosocial services, specifically, to the models of service delivery that are primarily individual-focused [13, 37–39]. According to the participants, there are capacity problems arising from how services are organized and planned. Services are not integrated fully and are not culturally contextualized in an adequate manner. The lack of a common approach, goals and theoretical underpinnings for the people sharing different cultural backgrounds may also contribute to the capacity barriers [40]. Thus, treatment cannot be easily extended to reach people who are not readily served by the prevailing models of service delivery. Raviola et al. [41], whose work focuses on the mental health treatment gap, conclude that “*task sharing*” expedited by service delivery models such a “*balanced care*” a systems-based framework, collaborative care and a stage transdiagnostic approach, all of which are facilitated by digital technologies, can be useful in bridging the gap.

The HPs who took part in the MentALLY project discussed ways to optimize service delivery, including integrated primary care, collaborative care, person-centered modes of care and community approaches. The literature and the participants in this study highlight that integrated care which includes co-location for health and MHS and a wide array of services from mental health literacy, to prevention, and person-centered and community-based care are means of making improvements and possible paths to providing effective care [42]. Integrated primary care combines medical, psychological, and social health care, where a team of professionals apply a systematic and systemic approach to the provision of services [37].

Participants discussed the vital role of integrated primary care since it is a gateway and a fundamental source of help for people seeking services. They explained that health service provision relies on an effective integrated primary care system. They specified that referrals and ensuring the links between the different levels of service delivery depend on a well-organized primary care system. For instance, the HPs in Norway and Sweden deliberated about the difficulties involved in making referrals and collaborating with colleagues and pointed out several barriers to referrals that have also been highlighted in the research literature. These include difficulties in collaboration between primary and specialized care, lack of specific ‘severity’ criteria and evaluation tools in primary care, and the increasing demand for mental healthcare services [43, 44]. As Beckers et al. [8] point out, making a referral is not an easy task given that several factors need to be taken into consideration, including the characteristics, support networks, and social situations of service-users. The lack of a clear path to treatment for those seeking services was emphasized in all of the focus groups. According to participants and the literature, the organization of care is like a maze without an accessible path between the different levels, resulting in mismatches in care provision [36]. In particular, HPs in Sweden discussed the challenges service users experience in accessing psychiatric care and the convoluted and time-consuming referral process and care pathway between primary healthcare and psychiatric care. According to the HPs such labyrinthine processes jeopardize service users’ safety and engagement.

Collaboration is a key ingredient in providing quality mental healthcare by the participants in this piece of research and in the recent literature [45–47]. Participants described how collaboration is not a simple process in that it involves working with people within different healthcare and social care systems and services. This is consistent with other studies that attribute collaboration difficulties to divergent values, communication, and efficient and accurate decision-making processes [48]. According to Reeves et al. [47], inter-professional care entails teamwork, collaboration, coordination and the development of networks with and across the different levels of care provision. These researchers maintain that such efforts require a shared identity, interdependence, integration and clarity of roles and goals. It seems that these processes are complex and difficult to bring to fruition.

Collaboration between HPs also entails the sharing of expertise and mentoring of inexperienced personnel [46]. Møller et al. [49] point out that organizational problems are barriers to collaborative care practices, as is the lack of clarity in purpose and in role requirements when working in interdisciplinary teams as well as difficulties in communicating and knowledge sharing.

The participants agreed that collaboration with service-users is an ethical issue in that people seeking health services have the right to voice their opinions, including the right to choose providers and to have access to quality care. Hence, service user involvement and co-production between HPs, service-users and their carers contributes substantially to optimal MHC provision because it entails respectful dialogue, sharing of insights, and mutuality, all of which the participants say enrich services and fortify positive relationships [45]. The HPs who contributed to the focus group discussions spoke adamantly as to how service-users are not numbers or labels and certainly do not want their humanity debased nor their freedom suppressed. Collaborative models in MHC delivery have been linked to quality care and benefits for HPs and people using healthcare services [45, 50, 51]. The all-important holistic, collaborative and person-centered approaches discussed in the literature are considered pathways towards adequately addressing the mhcGAP [7–9].

The third barrier discussed in the focus groups was care delivery. According to the HPs in all MentALLY countries, mental health problems are often complex and interdisciplinary collaboration is essential. Participants also underscored the complexities involved in assessing and providing services for mental health issues and the fine lines that exist between diagnosis, labeling, and stigmatization. In the focus groups in Norway and Sweden, the “*chase for diagnosis*” was a description that had negative connotations, including trepidation and “*eschewing*” such practices in order to better serve people in “*distress*”. Accordingly, Patel [52] calls attention to the rift that exists in communication with service-users and the community at large that is created by medical nomenclatures and typologies in diagnostic systems that professionals use as opposed to how *“distress”* occurs in people’s lives. Patel [52] also dubs this schism the “credibility gap” (p.16). He states, “a key problem lies in the gap between the understanding of mental disorder that mental health specialists use, best illustrated through the diagnostic systems and epidemiological instruments arising from them, and how the rest of the world conceptualizes psychological suffering.” (p. 16). The argument is against the medicalization of people’s emotional lives, labelling, and underemphasizing the impact of such practices on the people’s life worlds and on HPs’ abilities to understand and communicate with the communities they serve.

Patel’s [52] “credibility gap” was echoed in the focus group discussions, where according to the HPs, therapy practices have to be relevant to people’s everyday lives, and collaboration at different systemic levels is crucial in achieving this. Participants stressed how positive collaborative relationships are indispensable when HPs want to fit care practices to each service-user’s unique circumstances, to bolster relationship-building and to enhance therapeutic outcomes. Mental health service delivery is highly context-specific with culturally-defined interpretations of stigma, trust, and usefulness affecting favorable outcomes [10]. HPs also need to think in a systemic fashion, paying close attention to each person, their relationships and the social milieu, as these are all important elements of therapeutic interventions [37]. Moreover, such a context encompasses the community [39], rendering the community’s voice, needs and engagement indelibly connected to MHC. Developing systems and practices for inter-sectoral collaboration is considered a step toward establishing and fortifying optimal mental healthcare in communities around the world [42, 53].

Indeed, MH challenges emanate from a multitude of factors, including other health problems and contextual and sociocultural factors, all of which interact in a dynamic fashion [12]. This complexity and the concomitant ambivalence in defining mental health concerns are barriers to providing optimal care and create the need for continuous training, systemic evaluations of MH needs and flexibility in care provision since diagnosis alone can easily lead to labeling and stigmatization [54]. The participants in the focus groups discussed how stigma thwarts the connections between people and communities, rendering community-based care cumbersome [12, 37, 39]. The HPs in this study discussed ways to transverse this barrier and talked about the newly established mobile MHS and the use of mobile phones and e-mental health. These practices are substantiated in the research literature focusing on rural MHS [55]. E-mental health services were discussed in the focus groups in all six countries. The HPs from Greece, Norway and Cyprus advocated for such services due to geographical differences in availability and accessibility to service provision. In the other MentALLY countries e-mental health services were considered a solution to the overburdened primary healthcare systems. The HPs conclusions coincide with the international literature where e-mental health is considered to be an efficient, valid, and economic alternative for the provision of services across the continuum of MHC [56, 57]. For immigrant and refugee populations community-based care that is culturally-informed, holistic and person-centered care is recommended [58]. Person-centered and community-based approaches are also indicated for those with severe MH challenges [59].

Shared responsibility of both service-users and HPs, which was discussed by HPs in the Netherlands, would be far-reaching in the provision of quality services [39]. Likewise, HPs described how collaboration with service-users is integral in order to ensure therapy compliance and an effective therapy outcome (Cyprus, Belgium). Co-production that is mandated by policies was also deemed necessary in all the countries that took part in the research. Yet, as Stomski and Morrison [60] conclude in their meta-synthesis of studies focusing on co-production from service-users’ and HPs’ perspectives, up to now, service-users’ engagement in MHC remains a lofty goal on policy agendas. Barriers to implementing policy, as well as power imbalances between HPs and service-users, impede participation. Accordingly, quality care is based on trusting and mutually positive relationships between individuals seeking MH services and professionals [61].

The focus group participants championed comprehensive services that include the holistic understanding of peoples’ lives and continuity in care. The flexibility in how care is provided, comprehensive services, and forging positive and empowering relationships with people seeking services have also been found to contribute to more effective work in MHS [48]. They discussed the need for MH promotion strategies and for building the capacity of MHS to respond to mental healthcare needs with increased and more purposeful attention to people’s unique lives and circumstances (e.g., Norway). HPs also discussed psycho-education as part of more comprehensive and person-centered interventions since it is a structured educative approach that can inform people about the problems they are confronting, guide people in the prevention of complications or the development of problems and assist people in seeking or using services according to their self-defined goals and needs in order to obtain the outcomes they seek [62].

HPs described another aspect of optimal care as *“being authentically there”* and developing a positive and unique long-term relationship or ‘bond’ with service-users. They also emphasized that person-centered care should not be hindered by administrative constraints. According to Erskine [63] working collaboratively, fostering inter-subjective understanding and being authentically and congruently there in a compassionate manner are essential interpersonal components of MH interventions. According to Green et al. [59] person-centered care allows HPs to become partners with service-users, to express understanding and respond in a culturally mindful manner to the individual’s concerns. MHC can only be adequate when care providers treat people not as objects—diagnoses or parts of their problem—but as people with dignity. The suggestions and solutions the participants discussed provide insights into how to bridge the “accessibility–availability” and the “credibility” gaps in MHC provision. HPs described how to engage people within their community contexts in ways that can prove to be cost-effective (peer support, employment, and psychoeducation services) that can be useful strategies to keep people feeling engaged and supported.

## Limitations

Conducting multidisciplinary research across different countries that speak different languages is a challenge for qualitative researchers. In this study, the language and terminology used by the HPs in describing the organization and delivery of MHS across the different countries were not harmonized. The present study is also limited in terms of its transferability as it involved a small sample of HPs from each country involved in the project. Particularly concerning Belgium, participants were recruited in the Flanders region (and not from the French or the German-speaking regions). Potentially there are some cultural differences related to stigma and talking openly about mental health among these three regions, and hence some results are probably not representative for the whole of Belgium. Although much effort was put into representing the HPs as much and as accurately as possible, participants were a purposive, self-selected sample of HPs. Therefore, the findings may not reflect the experiences of the broader HP population in the six countries or HPs working within different healthcare structures. Limitations notwithstanding, participatory research across different countries leads to a greater understanding of cultural differences in service provision and in how professionals in different settings understand and implement strategies to ameliorate mental health problems.

## Conclusions

The qualitative data collected in this phase of the MentALLY project illuminated the barriers and facilitators in the provision of MHC in the six European Countries studied: Belgium, Sweden, Norway, the Netherlands, Greece, and Cyprus. The HPs who took part in this study are cognizant and apprehensive about existing care delivery and “credibility” gaps and discussed ways to make MHC relevant. The mhcGAP is comprised of the following barriers: funding, human resources capacity, accessibility to comprehensive services, and the availability of relevant services. The facilitators to the provision of MHC include the trustworthiness, reliability, and integrity of HPs. According to the participants, such capacities bolster the provision of pertinent, valid, and appropriate care. Likewise, participants highlighted collaborative models of primary, secondary, and prevention-oriented mental healthcare, which were deemed positive and vital in bridging the mhcGAP. Teamwork in providing care was considered to be more productive and efficient when it comes to the use of resources. HPs believe that the use of e-mental health and emerging digital technologies can enhance collaborative practices and can be used to provide access to care in hard to reach populations. Access to a continuum of community-based care that is responsive, coordinated, and in line with people’s needs throughout their lives constitutes another facilitator of optimal care. The diffusion of the knowledge generated from the MentALLY project is one of its core goals. The diffusion and transfer occurred by when policymakers from the European Commission followed and monitored the project (through outputs, meetings, and conference proceedings). Knowledge was and is being transferred when the general public, academics, mental health professionals, students and trainees, and service users and their networks participated in the focus group discussions, attended meetings and conferences, and took part in the massive online course (MOOC). The course is a freely available educational tool that will make evidence-based knowledge available for a wide range of stakeholders and practitioners.

Nevertheless, the authors acknowledge the tentative nature of the findings from this pilot study and the need for future collaborative efforts across Europe in order to develop a more in-depth understanding of how HPs conceptualize and approach the mental health concerns of the populations they serve, to develop frameworks for effective and accessible care and to apply such praxis, and to exchange tools and practices within networks of MHs, service users and their networks and communities. Future studies can apply quantitative and/or mixed methods methodologies and should include larger European samples of HPs, service-users, and policymakers to compare their views on issues of MHC provision. It is thus necessary to continue and broaden such research initiatives and to advocate for the enactment of comprehensive and holistic approaches at both the policy and service provision levels of care.

## Data Availability

The datasets (anonymized translated transcripts) generated and analyzed during the current study are available in the MentALLY Program repository.

## Abbreviations

HPs: Health professionals
HS: Health services
MHC: Mental healthcare
mhcGAP: Mental healthcare gap
MHS: Mental health services
PC: Primary care
TA: Thematic analysis
